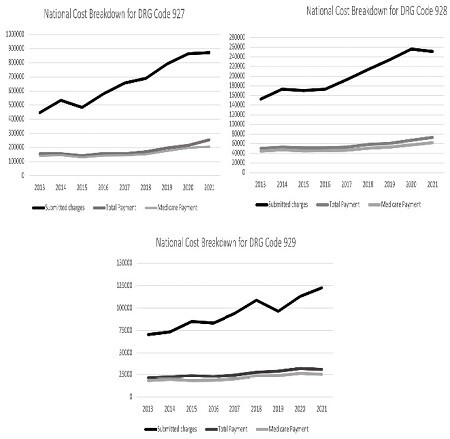# 535 An Analysis of Hospital Cost and Reimbursement for Burn Care : Are We Keeping Up?

**DOI:** 10.1093/jbcr/irae036.169

**Published:** 2024-04-17

**Authors:** Mariah Fleischman, Dhaval Bhavsar, Grace Jean

**Affiliations:** Creighton University, Omaha, NE; The University of Kansas Health System, Kansas City, KS; Touro University College of Osteopathic Medicine,, Las Vegas, NV; Creighton University, Omaha, NE; The University of Kansas Health System, Kansas City, KS; Touro University College of Osteopathic Medicine,, Las Vegas, NV; Creighton University, Omaha, NE; The University of Kansas Health System, Kansas City, KS; Touro University College of Osteopathic Medicine,, Las Vegas, NV

## Abstract

**Introduction:**

Hospital based care for burn patients is very resource intensive. Care of large surface area burn patients involve prolonged ICU stays, numerous surgeries, routine use of costly skin substitutes, almost daily wound care, longitudinal rehabilitation services. Discrepancies between hospital-related burn care costs and reimbursement can negatively impact financial health of burn centers. In this study, we evaluated trends in cost of burn care and reimbursement.

**Methods:**

We reviewed available data from Center for Medicare and Medicaid Services (CMS) for years 2013-2021. The information obtained included submitted total hospital charges, total payments, and hospital payment for DRG (disease related group) codes 9127, 928, and 929 (related to burn care). Additionally, same data was collected for DRG codes related to Bronchitis, Heart Failure, and skin ulcer. Burn associated cost of care and reimbursement was compared against skin ulcer wounds, heart failure, and bronchitis to determine funding variance between different diagnoses. Rate of increase in cost of providing burn care and CMS reimbursement were compared. These changes were also compared to annualized inflation data available through Department of Labor.

**Results:**

We observed trend of increasing burn care costs as evidenced by submitted charges from 2013-2021. Year to year increase for submitted charges was nearly 12%. Reimbursement for burn care increased over the same period too though this increase lagged the increase in cost of care. Year to year increase for total medicare reimbursement was nearly 8% over 2013-2021. Increase in CMS reimbursement was higher than the anticipated numbers based on inflation adjustment alone though lagged far behind real increase in cost of care. Reimbursement data for Skin ulcer, but not Bronchitis and Heart Failure, followed similar trend. Results are tabulated in attached graph.

**Conclusions:**

Cost of providing burn care has increased significantly over the observed period of 2013-2021. Though reimbursements have increased also during this time, they have not kept up with increase in cost of care. This discrepancy suggests burn centers may be experiencing worsening financial stress. While healthcare costs increase annually due to inflation, the greater incremental increase in burn and skin ulcer wound care compared to other conditions reflects a financial demand that cannot be due to inflation alone.

**Applicability of Research to Practice:**

Understanding healthcare economics related to burn care may help us run improve fiscal health of burn centers.